# Sensitivity Enhancement of a Surface Plasmon Resonance with Tin Selenide (SnSe) Allotropes

**DOI:** 10.3390/s19010173

**Published:** 2019-01-05

**Authors:** Xiaoyu Dai, Yanzhao Liang, Yuting Zhao, Shuaiwen Gan, Yue Jia, Yuanjiang Xiang

**Affiliations:** 1 International Collaborative Laboratory of 2D Materials for Optoelectronic Science & Technoloy of Ministry of Education, College of Optoelectronic Engineering, Shenzhen University, Shenzhen 518060, China; xiaoyudai@126.com (X.D.); 1810285030@email.szu.edu.cn (Y.L.); 2172281521@email.szu.edu.cn (Y.Z.); 2172281559@email.szu.edu.cn (S.G.); xiangyuanjiang@126.com (Y.X.); 2 Laboratory of Advanced Material Photonics (LAMPs), Shenzhen University, Shenzhen 518060, China

**Keywords:** SnSe allotrope, SPR sensor, high sensitivity

## Abstract

Single layers of tin selenide (SnSe), which have a similar structure as graphene and phosphorene, also show excellent optoelectronic properties, and have received much attention as a two-dimensional (2D) material beyond other 2D material family members. Surface plasmon resonance (SPR) sensors based on three monolayer SnSe allotropes are investigated with the transfer matrix method. The simulated results have indicated that the proposed SnSe-containing biochemical sensors are suitable to detect different types of analytes. Compared with the conventional Ag-only film biochemical sensor whose sensitivity is 116°/RIU, the sensitivities of these SnSe-based biochemical sensors containing α-SnSe, δ-SnSe, ε-SnSe, were obviously increased to 178°/RIU, 156°/RIU and 154°/RIU, respectively. The diverse biosensor sensitivities achieved with these three SnSe allotropes suggest that these 2D materials can adjust SPR sensor properties.

## 1. Introduction

Due to their high sensitivity, biocompatibility and detection accuracy, biochemical sensors based on the surface plasmon resonance technique have been investigated and applied in numerous chemical and biological applications [1], for instance, medical diagnostics, enzyme detection, and pharmaceutics [2,3,4]. The SPR phenomenon refers to the resonance excitation of the surface plasmon polaritons (SPPs) at the interface of negative (metal) and positive (dielectric) constant materials [5], which is suitable for label-free sensing and the real-time monitoring [6].

One of the best ways for making sensing devices is to choose a large surface area binding/adsorbing material, like the popular 2D material graphene. Transition metal dichalcogenide (TMDC) materials, which have excellent properties such as an absorption rate (~5%) which is higher than that of monolayer graphene (2.3%), a large tunable band gap which is quite different from the zero band gap of graphene, and large work functions as compared with those of graphene, are becoming the preferred materials among the biosensing research fraternity. The fantastic optical, electrical, chemical properties of TMDCs make them promising candidates and successful materials compared to graphene for the next generation of optic and electronic devices [7,8,9]. TMDC-based SPR sensors which exhibit enhanced sensitivity have been proposed for refractive index sensing [10,11].

Single layer SnSe also possesses fantastic electrical and optical properties and generates high interest as a 2D material beyond the predecessor members, which displays the same structure as graphene and phosphorene [12,13]. As a classical p-type IV–VI semiconductor, SnSe has a narrow gap (~1.30 eV direct and ~0.90 eV indirect), and additionally, it also shows less toxicity, and better chemical stability. It is well known that SnSe has a layered crystal structure similar to those of other IV–VII binary semiconductors, such as SnS, GeS and GeSe, etc. In experiments, the assumption that unilaminar crystal SnSe would display different thermoelectric properties on different axes was authenticated by Zhao et al. [14], who manufactured hole-doped single crystal SnSe [15]. A lot of SnSe allotropes have been investigated by density functional theory [16]. Monolayer SnSe not only has outstanding thermoelectric features with semiconductor properties, but is also an eminent optoelectronic material, but there are few systematic studies on the optic and electric characteristics of SnSe allotropes.

As we know, Au is not susceptible to oxidation and does not react with most chemicals, and hence it is often used as the metal film in sensors. In practical applications, Au films can be replaced by other metals like Ag, which has been found to have higher sensitivity than Au in biosensor applications. However, Ag has poor resistance to oxidation in many environments. In order to protect Ag from oxidation and increase biomolecule adsorption, we introduce SnSe. Additionally, SnSe can also be used as the protective layer adjoined the biomolecular recognition elements to prevent oxidation, increasing the biomolecule adsorption. Therefore, we can use an Ag-SnSe structure to replace the Au film in sensors to obtain new biosensors. In this paper, we have introduced the 2D material SnSe to a type of SPR biochemical sensors to improve their sensitivity and maintain their chemical stability. A Kretschmann attenuated total reflection (ATR) configuration was chosen as these SPR sensor structure. In this structure, a thin metallic silver (Ag) layer was covered on the basis of the optical coupling prism, BK7 glass was used as coupling prism and the Ag layer was separately covered with three types of SnSe allotrope [17,18].

## 2. Calculation Models and Methods

**Figure 1:**
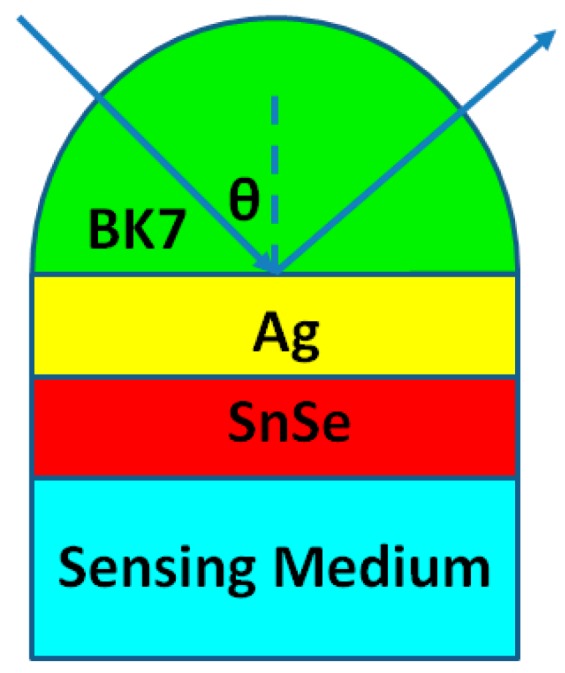
Sketch diagram of the SPR biochemical sensor made using three types of SnSe allotropes to enhance the sensitivity.

The structure of the proposed high sensitivity sensor separately containing three types of SnSe allotropes is shown in Figure 1. The thickness of the SnSe monolayer is 0.575 nm [19]. BK7 glass is used as coupling prism; silver film used as the first-rank metal for SPP is selected from 48 nm to 52 nm. The BK7 glass refractive index is calculated from Equation (1) [20]:

(1) nBK7=(1.03961212λ2λ2−0.00600069867+0.231792344λ2λ2−0.0200179144+1.03961212λ2λ2−103.560653)12

The refractive index of silver (Ag) can be expressed by the Drude-Lorentz model [21]:

(2) nm=εm=[1−λ2λcλp2(λc+iλ)]12

where *λ_c_* and *λ_p_* are the collision and plasma wavelengths, and the numerical values of *λ_c_* and *λ_p_* for Ag are 1.7614 × 10^−5^ m and 1.4541 × 10^−7^ m, respectively. SnSe is covered on the Ag metal film surface to impede the metal from being oxidized, which farther increases the sensibility of the designed biochemical sensor.

**Figure 2:**
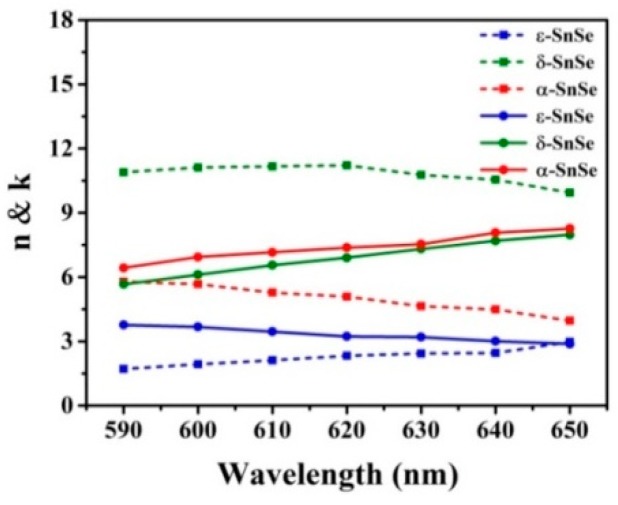
Measured refractive indices for the three SnSe allotropes [22].

The refractive indices, as variable quantities of the three SnSe allotropes and composed of a real part and an imaginary part are obtained from the experimental data of Cui et al. [22]. Figure 2 shows the refractive index of these three SnSe allotropes at wavelengths from 590 nm to 650 nm, where inside the full curves are the real parts (*n*) and the dotted lines are the imaginary part (*k*) of the SnSe allotropes. At *λ* = 633 nm, the refractive indexes of the three SnSe allotropes are 8.59 + 4.03*i*, 9.94 + 9.2*i*, and 4.4 + 3.53*i*, respectively.

The expression *n_s_* = 1.33 + Δ*n* is the refractive index in the sensitive medium, and Δ*n* is the change of refractive index in the sensitive medium causing by chemical reactions or biological actions.

We have employed the transfer matrix method [23] to evaluate the reflectivity of the incident TM-polarized light of the *N*-layer model, because the matrix method is pinpoint and without approximations. In these SnSe allotrope biosensors, all layers are piled in the direction of the vertical BK7 glass coupling prism, and each layer is represented by a dielectric constant (*ε_k_*), refractive index (*n_k_*), and thickness (*d_k_*), respectively. The resonance angle is defined of the minimum reflectance corresponding to the incident angle. The tangential field of the first boundary *Z* = *Z_1_* = 0 is correlated with the tangential field of the final boundary *Z* = *Z_N_*_−1_:

(3) $$\left[\begin{array}{c} U_{1}\\ V_{1} \end{array}\right]=M\left[\begin{array}{c} U_{N-1}\\ V_{N-1} \end{array}\right]$$

where *V* and *U* delegates the ingredients of magnetic and electric fields at the limiting surface. *M* is the characteristic matrix of the composite architecture, and *k* is the *k*-th layer in the *N*-layered model, and for *p*-polarized light it is made up of:

(4) $$\prod {}_{K=2}^{N-1}M_{K}=\left[\begin{array}{cc} M_{11} & M_{12}\\ M_{21} & M_{22} \end{array}\right]$$

(5) $$M_{K}=\left[\begin{array}{cc} \cos\beta_{K} & \frac{-\sin\beta_{K}}{q_{k}}\\ -{\mathrm{iq}}_{k}\sin\beta_{k} & \cos\beta_{k} \end{array}\right]$$

(6) qk=(μkδk)12cosθk=(εk−n12sinθ12)12εk

(7) βk=2πλnkcosθk(Zk−Zk−1)=2πdkλ(εk−n12sinθ12)12

After some straightforward mathematical steps, we can obtain the p-polarized light *N*-layer complex reflection coefficient *r_p_*, and the corresponding amplitude reflection coefficient (*R_p_*) can be obtained by the square of *r_p_*:

(8) $$r_{p}=\frac{\left(M_{11}+M_{12}q_{5}\right)q_{1}-\left(M_{21}+M_{22}q_{5}\right)}{\left(M_{11}+M_{12}q_{5}\right)q_{1}+\left(M_{21}+M_{22}q_{5}\right)}$$

(9) $$R_{p}={\left|r_{p}\right|}^{2}$$

The transformation of sensing medium in refractive index (Δ*n*) can initiate the change of resonance angle (Δ*θ*), and the sensitivity can be indicated as SR1 = Δ*θ*/*Δn* [24].

## 3. Results and Discussion

**Figure 3:**
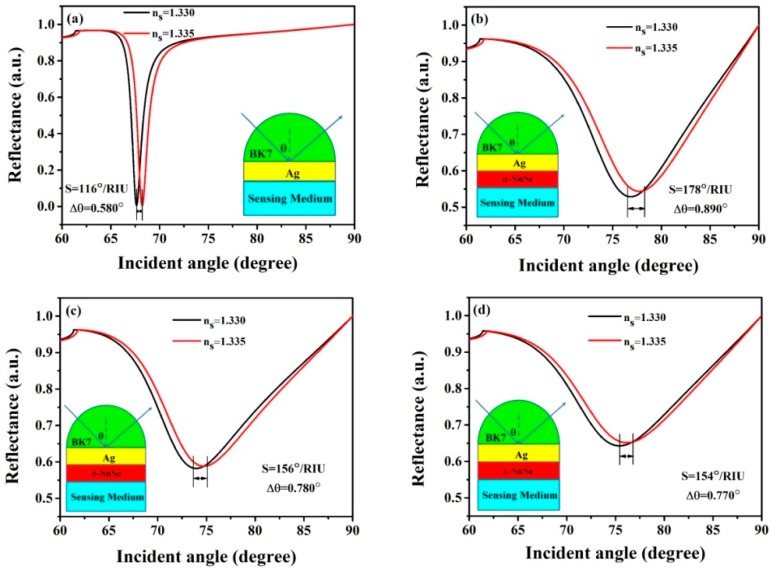
Variation of reflectivity with incidence angle for (**a**) the conventional biochemical sensor based on a simplex Ag film, (**b**–**d**) are the proposed biochemical sensors with α-SnSe, δ-SnSe, ε-SnSe, respectively.

BK7 glass has a low refractive index, which is a beneficial property for its use as a coupling prism in these expected biochemical sensors. In the architecture displayed in Figure 3a, the Kretschmann geometry-based conventional SPR biochemical sensor always employ a simplex metallic layer to motivate and survey SPP. The sensitivity of the conventional mental Ag-based SPR biochemical sensor is 116°/RIU (Figure 3a) when utilizing a BK7 glass prism. This value is not satisfactory for biochemical sensor sensitivity [2,25]. In this work, we designed SPR biochemical sensors [26,27,28] by utilizing three types of 2D material TMDC SnSe allotropes to improve the sensitivities of sensors.

Three types of 2D TMDC material SnSe allotropes have been added in the biosensor structure between the Ag and the sensing medium, respectively, to enhance the sensitivity and to protect the metal Ag from being oxidized. The results show that the sensitivities displaye a large enhancement by applying a monolayer of SnSe upon the former structure, as displayed in Figure 3b–d. Sensitivities of up to 178°/RIU, 156°/RIU, 154°/RIU after accumulating 15, 9, 13 layers are obtained separately.

**Figure 4:**
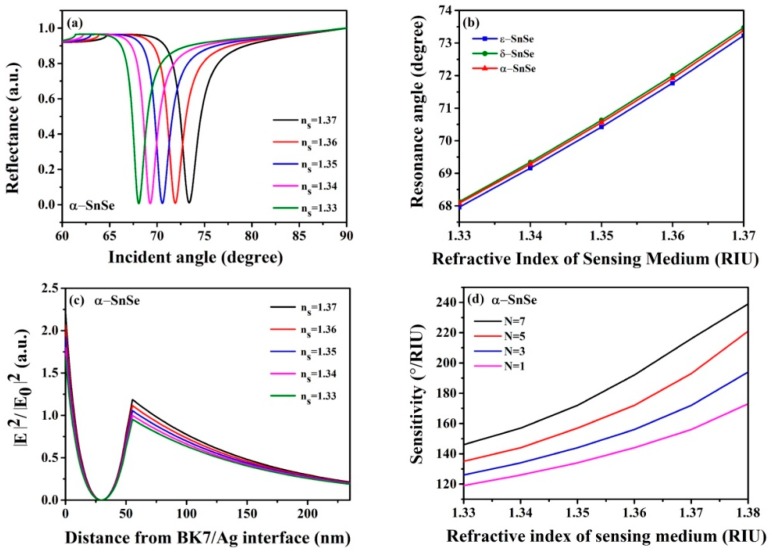
(**a**) Change of reflectivity relative to incidence angles, (**b**) the electric field distributions of the biochemical sensors proposed by SnSe from *n_s_* = 1.33 to *n_s_* = 1.37, (**c**) incidence angles of three SnSe allotropes from *n_s_* = 1.33 to *n_s_* = 1.37, (**d**) the variation of the sensitivities of the proposed biochemical sensors with different number of SnSe layers with the refractive indices of the sensing medium.

The changes of reflectivity with the incidence angle are drawn in Figure 4a. It can be seen that the resonance angles of α-SnSe sensor with one layer shifts to a greater incidence angle with the increase of refractive index of the sensitive media. The resonance angles are 68.08°, 69.28°, 70.56°, 71.92° and 73.39° when *n_s_* = 1.33, *n_s_* = 1.34, *n_s_* = 1.35, *n_s_* = 1.36 and *n_s_* = 1.37, respectively. Allotrope δ-SnSe, ε-SnSe containing sensors also have the same trend, and Figure 4b shows the resonance angles of the three SnSe allotrope biochemical sensors as the refractive indices of the sensitive media change from *n_s_* =1.33 to *n_s_* =1.37. The electric field distributions of the α-SnSe-based biochemical sensors when the refractive indices of the inductive media vary between 1.33 and 1.37 are shown in Figure 4. The electric field of the graphene/sensing medium interface changes obviously, while the other two allotropes of SnSe display the same phenomenon. This means that the designed SnSe-based biochemical sensors are sensitive to minor changes in sensitive media. The minor changes of refractive index of the sensitive medium near the interface will result in a great change of the surface wave characteristics, and a subsequent change of the electric field. Figure 4d the shows sensitivity change with different layers of α-SnSe based biochemical sensor from *n_s_* =1.33 to *n_s_* =1.38 in response to the sensing medium refractive index.

**Figure 5:**
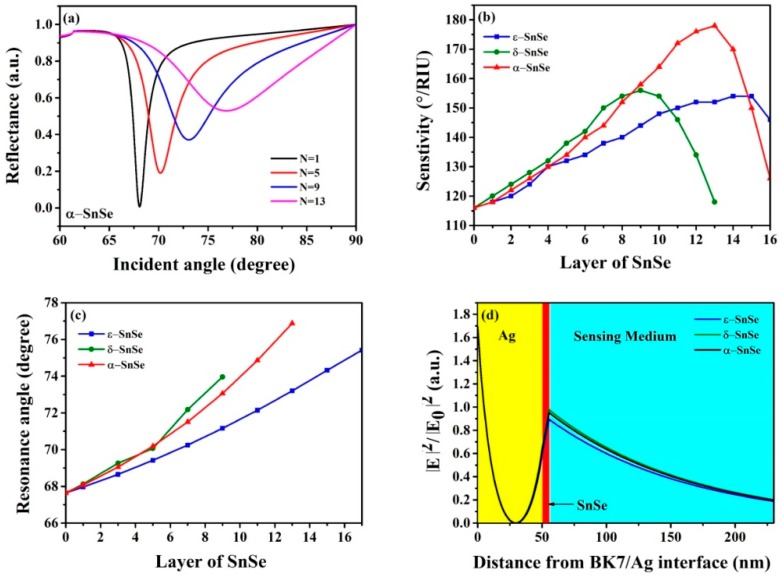
(**a**) Change of the reflectance of α-SnSe biosensor, (**b**) variation of sensitivity with respect to different numbers of SnSe allotrope layers, (**c**) change of resonance angle with respect to different layer numbers of SnSe allotropes, (**d**) the electric field distribution for the proposed SPR sensor.

2D TMDC material SnSe allotrope-based biochemical sensors show enhanced sensitivities. Figure 5a shows that the reflectivity of α-SnSe layers varies with the incidence angle. Figure 5b gives the sensitivity changes of these three SnSe allotropes on the Ag surface. The results show that the highest sensitivities are 178°/RIU, 156°/RIU and 154°/RIU for the α-SnSe, δ-SnSe, ε-SnSe SnSe allotropes, respectively. Figure 5c is the change of resonance angle for the three SnSe allotropes by different number of layers. Figure 5d exhibits the electric-field distributions for the proposed SPR sensors with three SnSe allotropes, respectively. It is found that coating SnSe on the Ag film can improve the electric field at the sensor/sensing medium interface, with δ-SnSe reaching the highest electric field strength, α-SnSe being in the middle and ε-SnSe displaying the lowest for a single layer of SnSe.

Table 1 give the detailed information about Ag thickness (*d_Ag_*), optimized layers (*L*), change in resonance angle (Δ*θ*), minimum resonance angle (*θ_min_*), the smallest reflectivity (*R_min_*) and the highest sensitivities to these SnSe allotrope biochemical sensors. The incidence angle range is set from 0° to 90°. The thicknesses of the SnSe layers are calculated by overlying the thickness of monolayer SnSe which is 0.575 nm. By increasing of SnSe layer number, the reflectivity curve moves forward at a high angle. We can obtain the highest sensitivity when the layers of SnSe increase to the optimized value. The sensitivities reach the maximum when the accumulated layers are 13, 9 and 15, respectively, for α-SnSe, δ-SnSe and ε-SnSe. 

After exceeding the optimized number of SnSe allotrope layers, due to the limitation of the angle range, the variation of the resonance angle will reduce and the sensitivity will begin to decrease too.

## 4. Conclusions

In this paper, we have designed and emulated SPR biochemical sensors utilizing three 2D TMDC SnSe allotropes separately to enhance the sensitivities, and the different characteristics resulting from the use of these three SnSe allotropes have been discussed. SnSe allotropes are layered on Ag films to improve the sensor sensitivities and also as protective layers, and it is shown that they improve the sensitivities in the devised biochemical sensors to 178°/RIU, 156°/RIU and 154°/RIU for α-SnSe, δ-SnSe, ε-SnSe, respectively.

## Figures and Tables

**Table 1:** Change in different Ag thickness (*d_Ag_*), layer numbers (*L*), highest sensitivity (*S*), minimum resonance angle (*θ_min_*), resonance angle change (Δ*θ*), and the smallest reflectivity (*R_min_*) for the SnSe allotrope-based SPR biochemical sensors at *n_s_* = 1.33.

SnSe Allotropes	*d_Ag_* (nm)	*L*	*S* (°/RIU)	*θ_min_* (Degree)	Δ*θ* (Degree)	*R_min_* (a. u.)
without SnSe	52	0	116	67.64	0.58	0.02690
α-SnSe	52	1	118	68.08	0.59	0.00003
	52	8	152	72.27	0.76	0.3954
	52	13	178	76.91	0.89	0.5854
δ-SnSe	52	17	118	68.14	0.59	0.01282
	52	5	138	70.60	0.69	0.4446
	52	9	156	73.98	0.78	0.6327
ε-SnSe	52	13	118	67.96	0.59	0.00100
	52	8	138	70.70	0.69	0.4513
	52	15	156	74.33	0.78	0.6476
without SnSe	50	0	116	67.64	0.58	0.00590
α-SnSe	50	1	118	68.08	0.59	0.00680
	50	8	152	72.25	0.76	0.3295
	50	13	178	76.86	0.89	0.5288
δ-SnSe	50	17	120	68.13	0.60	0.03950
	50	5	138	70.59	0.69	0.3805
	50	9	156	73.95	0.78	0.5821
ε-SnSe	50	13	118	67.96	0.59	0.01420
	50	8	138	70.69	0.69	0.3875
	50	15	154	74.31	0.77	0.5987
without SnSe	48	0	116	67.64	0.58	0.02691
α-SnSe	48	1	118	68.08	0.59	0.00003
	48	8	150	72.23	0.75	0.2632
	48	13	176	76.80	0.88	0.4673
δ-SnSe	48	17	118	68.14	0.59	0.01281
	48	5	138	70.58	0.69	0.3146
	48	9	154	73.92	0.77	0.5264
ε-SnSe	48	13	118	67.96	0.59	0.00103
	48	8	136	70.68	0.68	0.3218
	48	15	156	74.27	0.78	0.5447
